# Antibiotic use in post-adenotonsillectomy morbidity: a randomized prospective study

**DOI:** 10.1016/S1808-8694(15)30565-6

**Published:** 2015-10-19

**Authors:** Marja Michelin Guerra, Eduardo Garcia, Renata Ribeiro de Mendonça Pilan, Priscila Bogar Rapoport, Caio Barbosa Campanholo, Eli Onivaldo Martinelli

**Affiliations:** 1 Third-year Otorhinolaryngology Resident at FMABC.; 2 Sixth-year Student at the ABC Medical School.; 3 Fellow on intranasal endoscopic surgery at USP.; 4 Full Professor - Otorhinolaryngology Department at the ABC Medical School.; 5 Third-year Otorhinolaryngology Resident at FMABC.; 6 Third-year Otorhinolaryngology Resident at FMABC ABC Medical School.

**Keywords:** antibiotics, morbidity, tonsillectomy

## Abstract

Tonsillectomy with or without adenoidectomy still is one of the most commonly performed surgical procedures in the world, mostly in the pediatric population.

**Aim:**

to study the impact of amoxicillin for 7 days in post-adenotonsillectomy recovery, comparing results with a control group. Study type: prospective, randomized, controlled study with 120 patients.

**Patients and Methods:**

the patients were randomized according to surgery time to receive 7 days of amoxicillin associated with pain killers, or analgesic alone. During the first week of postoperative, we assessed the level of pain, oral intake acceptance, nausea and vomits, fever and return to daily activities.

**Results:**

It was only in the fourth postoperative day that the group receiving antibiotic agents showed a statistically significant difference as far as pain is concerned. There was no difference between the two groups for other data analyzed.

**Conclusion:**

considering the results from our study and reviewing the literature on the use of antibiotic agents, we agree that there is no improvement in patient recovery after adenotonsillectomy with the use of amoxicillin for 7 days in the postoperative.

## INTRODUCTION

Tonsillectomy, whether or not combined with adenoidectomy, still is one of the most frequently performed surgical procedures in the world, with higher prevalence among the pediatric population.^1^, ^2^ In the early 20th century this was a procedure medical doctors often resorted to, regardless of how minimally symptomatic patients were. Since the 1960s, however, there was marked progress in immunology and increased knowledge on the physiology and relevance of the Waldeyer's ring, which led to a review of the established indications for surgery. This procedure has since then been more judiciously indicated, with proper consideration of the patients’ clinical symptoms. Adenotonsillectomy, however, still is one of the most frequently performed surgical procedures within the realm of otorhinolaryngology.^3^

The main current indications for tonsillectomy and adenoidectomy are recurring infections and blockages of the upper airways, which may possibly lead to serous otitis, repetition otitis media, rhinosinusitis, snoring, sleep apnea, and altered craniofacial growth - often compromising the child's development and performance at school.^3^

There have been great developments in the fields of surgical technique and anesthesia, but post-operative morbidity is a yet important factor to be considered.^1^ Several papers have looked into drugs to minimize post-op morbidity, such as oral steroids administered during anesthesia or dosed regressively for the first days of post-op, analgesics such as paracetamol, diclofenac and tramadol, and prophylactic antibiotics.

Although some studies have shown antibiotics to be beneficial in reducing post-adenotonsillectomy morbidity, there is still no consensus on the matter. Part of the disagreement arises from the lack of well-designed clinical trials.

In 1980 Telian et al.^4^ showed the benefits of using ampicillin/amoxicillin in mitigating post-op symptoms such as fever and pain. In 1999 Colreavy looked at post-op pain reduction through the use of analgesics and verified a statistically significant improvement in the group of patients taking amoxicillin + clavulanic acid when compared to those in the control group. However, Al-Kindy, in 2001^5^, in a retrospective study comprising 185 patients could not support the use of antibiotics in this setting.

Specifically in terms of bleeding, there is very little clinical evidence to support the use of post-op antibiotics in reducing hemorrhage. A retrospective study by Ranjit et al.6 including children who underwent adenotonsillectomy and a study by O’Reilly et al. with adult patients7 showed that post-op antibiotics did not reduce the incidence of bleeding episodes.

Given such context, it is only timely that we carry out a prospective randomized trial in our service comprising patients who underwent adenotonsillectomy so as to assess the effect of administering antibiotics upon post-operative morbidity.

## OBJECTIVE

This paper aims to verify the role antibiotics have in the treatment of post-operative morbidity of adenotonsillectomy patients looking at the following parameters: pain, use of analgesics, bleeding, fever, vomiting, oral intake, and return to routine daily activity. The results will be compared against those of a control group.

## MATERIALS AND METHODS

This prospective randomized controlled trial was approved by the Medical Ethics Committee under permit 215/2005, and was conducted at a tertiary care hospital where patients underwent adenotonsillectomy in the first semester of 2006. Enrolled patients were 14 and under with surgical indication for adenotonsillectomy. Patients allergic to amoxicillin and with blood dyscrasia were taken off the study. The persons legally responsible for the patients were given medical clarification and signed a free informed consent term.

The 120 patients enrolled in the trial were divided into two groups: I. Intervention Group: treated in the post-op with amoxicillin 50mg/kg/day for seven days + symptom management drugs (paracetamol or dipyrone, dimenhydrinate if needed); II. Control Group: symptom management drugs when needed.

The surgical procedures were carried out by the otorhinolaryngology residents under the supervision of the attending assistant surgeon. The technique of choice was dissection, without the aid of the electrocautery.

The persons legally responsible for the patients were given a questionnaire to be answered daily for seven days into the post-op in which pain, presence of bleeding, fever, vomiting, dietary compliance, and return to routine daily activity had to be rated.

Twenty-five patients were excluded from the trial for not having handed in their questionnaires or having abandoned their post-op follow-up. The trail was thus completed with 95 patients.

Statistical analysis: descriptive analysis was done for all variables studied in the trial. Qualitative variables were presented in terms of their absolute and relative values. Quantitative variables were presented in terms of their central tendency and scatter values. The Kolmogorov-Smirnov and Levene tests were used to check the normality and homogeneity of variances respectively. The variables that passed both tests were submitted to the T test, while the ones that did not were submitted to the U Mann-Whitney test. The chi-square test was used in order to verify the associations between groups and genders. The level of significance was 5% (p<0.05). Statistical package SPSS 14.0 for Windows was used for all pertaining calculations.

## RESULTS

From the 95 patients enrolled in the trial, 43 belonged to Group 1 (intervention) and 52 to Group 2 (control). Both groups had similar age and gender distributions. Average ages were of 7.8 years (STD 3.16) in group 1 and 6.19 (STD 2.39) in group 2. In the intervention group, 55.8% of the patients were females and 44.2% males. In the control group, 51.9% were females and 48.1% males. There was no statistically significant difference in the pre-op status of the groups.

**Figure 1:**
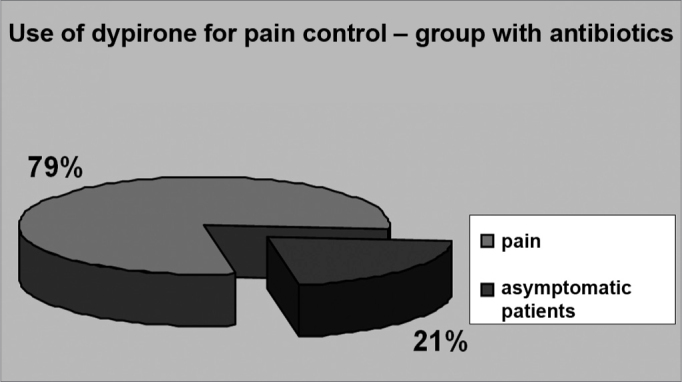
PAIN - Comparative chart - prevalence of pain between groups

There was no statistically significant difference in post-op pain among the groups, except on the fourth day of post-op, when pain levels in group 1 were significantly lower than in group 2 (p < 0.05) (Figure 1). For the seven days consecutive to surgery, pain levels were daily assessed in a scale where 1 meant painless and 5 meant very painful. The average pain level on the fourth day of post-op in group 1 was 1.86, whereas in group 2 that same variable amounted to 2.52. Pain was worse in group 1 between the first and third days of post-op, and between the first and fourth days of post-op in group 2.

**Figure 2:**
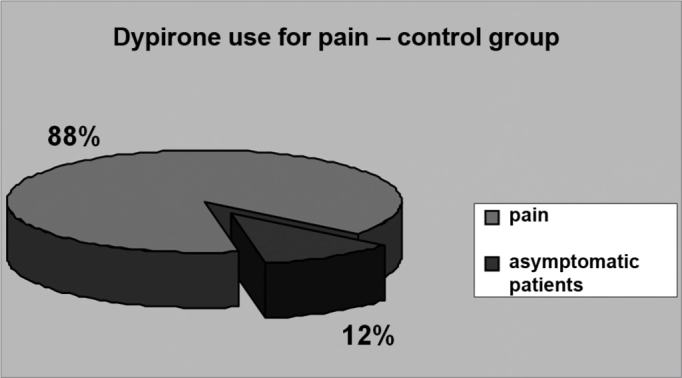
USE OF DIPYRONE TO MANAGE PAIN - GROUP 1 - 79% of the patients in group 1 required analgesia with dipyrone. Analgesics were mostly required by the second day of post-op and were administered to 23.3% of the patients

**Figure 3:**
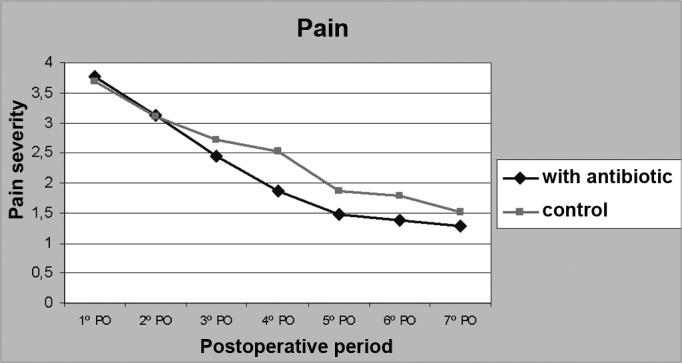
USE OF DIPYRONE TO MANAGE PAIN - GROUP 2 - 88% of the patients in group 2 required analgesics to manage their pain. Analgesics were mostly required by the third day of post-op and were administered to 21% of the patients.

Analgesics were mostly needed by the second day of post-op among group 1 patients (23.3%) (Figure 2) and by the third day of post-op among group 2 patients (21%) (Figure 3). The prescribed analgesic medication was used by 14% of all patients until the sixth day of post-op. None of the patients required additional analgesics or had to tend to an emergency unit due to refractory pain.

**Figure 4:**
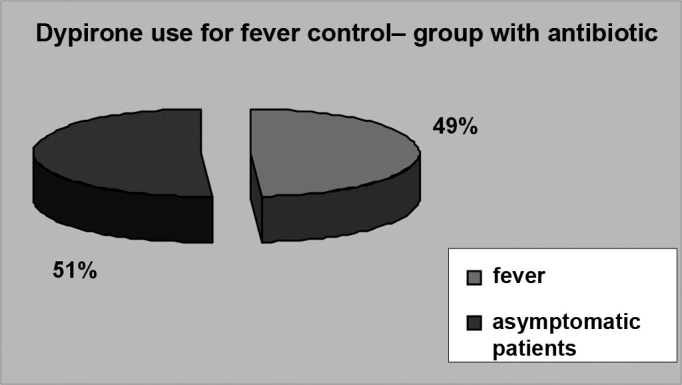
FEVER IN THE GROUP TREATED WITH ANTIBIOTICS - twenty-two patients (51.2%) in group 1 had fever in the post-op.

**Figure 5:**
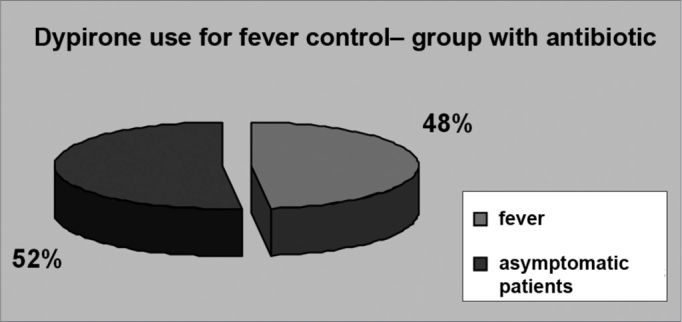
FEVER IN THE CONTROL GROUP - 26.9 patients (51.9%) in group 2 had fever in the post-op.

Even though fever is a common symptom in post-adenotonsillectomy pediatric patients, most of them did not have fever in the post-operative - 22 patients (51.2%) in group 1 (Figure 4) and 26.9 (51.9%) in group 2 (Figure 5). In the intervention group, 11 patients (25.6%) had fever until the second day of post-op, whereas in the control group 10 patients (19.2%) had fever also until the second day of post-op.

**Figure 6:**
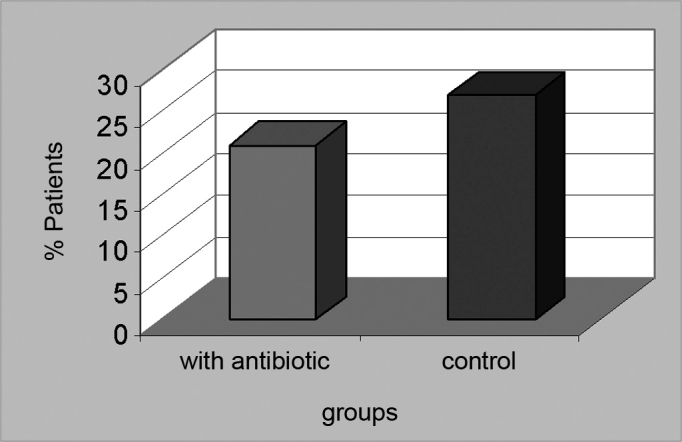
BLEEDING - incidence of post-op bleeding in the groups. No cases of primary bleeding were reported.

There were no cases of primary bleeding among trial patients. In group 1, nine patients (21%) presented secondary bleeding between the first and third days of post-op but did not require medical intervention. They were managed with local care. In group 2, fourteen patients (26.8%) presented secondary bleeding between the first and fifth days of post-op, and did not require medical intervention. There was no statistically significant difference between the groups (Figure 6).

Nausea and vomiting were reported by 12 patients (27.9%) in group 1. Five of these patients (11%) had such adverse effects in the first day of post-op. Thirty-nine patients (75%) from group 2 reported no nausea or vomiting. Eight (15.4%) of the patients reporting the discomfort presented nausea and vomiting in the first day of post-op.

Patients in group 1 took 4.14 days (STD 1.79) to go back to their normal diet while patients in group 2 took 4.12 days (STD 1.82). Patients in the intervention group took 4.58 days (STD 1.57) to return to their routine daily activity while patients in the control group needed 4.58 days (STD 1.86).

## DISCUSSION

Adenotonsillectomy still remains as one of the most frequent surgical procedures done in the pediatric population^2^. Morbidity in the post-op is mostly due to pain, fever, vomiting, bleeding, and fatigue, to name a few^5^. According to Telian et al.^4^, the use of antibiotics in the post-op improves various morbidity factors when compared to a placebo drug regimen. The authors have observed lower incidences of fever, earlier return to normal dieting and routine daily activity, and reduced pain in the group treated with amoxicillin^4^. The paper by Lee et al.^8^ however, showed that patients given post-adenotonsillectomy antibiotics did not present fewer morbidity factors when compared to the control group.

Pain in the post-op is worse during the first five days, and although many treatment options have been studied, none was consistently successful^9^, ^10^, ^11^, ^12^, ^13^, ^14^. In 2005, Collin et al.^1^ carried out a meta-analysis looking at randomized trials published in the literature and could not find statistically significant differences in post-op pain levels of adenotonsillectomy patients treated or not with antibiotics. Our study pointed to a statistically significant difference (p < 0.05) in the levels of pain of both groups in the fourth day of post-op, considering the first seven days consecutive to the procedure. Such improvement on pain levels in the fourth day of post-op is advantageous, as it favors earlier patient clinical recovery. Besides, any measure taken to successfully reduce patient post-op discomfort and mitigate parental anxiety is worth considering. Pain alleviation may prevent dehydration, mainly among younger children, and consequent hospitalization - often a traumatic experience for children and parents.4 The patients in our trial did not require any medication other than the prescribed analgesics to manage their pain. Talbot^15^ postulates that pain and sialorrhea in the post-op are more related to spasms in the pharyngeal constrictor muscles than to exposure of nerve endings.

Bleeding is another frequent post-operative complication^5^, ^7^. It was observed between the first and fifth days of post-op in 21% of the patients in the group treated with amoxicillin and in 26.8% of the patients included in the control group. However, it was expected that the patients’ caretakers would have difficulties in properly characterizing bleeding episodes. As none of the children went to the emergency unit nor required medical intervention, it was assumed that the reported episodes were of minor bleeding (defined as bleeding episodes taking place within 24 hours or surgery and reported by the patient or caretaker requiring no specific treatment)^7^. When looking at post-adenotonsillectomy complications and the need to see a physician, Terence et al.^9^ found an incidence of 2-4% of secondary bleeding, a low rate as considered by the authors when compared to other studies. This shows that the majority of the episodes characterized as bleeding by the patients’ caretakers do not require clinical intervention.

This study has also shown that the prevalence of nausea, vomiting, and fever, and the time required for patients to return to routine daily activity and oral intake were similar between the groups. However, according to Collin et al.^1^, patients taking antibiotics in the post-op returned one day earlier to their daily routine activities and to oral intake, presenting a statistically significant difference against untreated patients. Telian et al.^4^ pointed to a reduction in the prevalence, duration, and severity of fever episodes among patients treated post-operatively with antibiotics. These patients were also able to return earlier to their daily routine activities and regular feeding habits. This is probably advantageous in the treatment of younger children, as fewer of them will require hospitalization due to dehydration and consequent intravenous rehydration.

## CONCLUSION

Although antibiotics were deemed beneficial by some studies in relation to post-adenotonsillectomy morbidity^1^, ^4^, one needs to consider the significant prevalence of adverse effects in the patient population^1^, ^16^ and, above all, the issue of bacterial resistance to antibiotics and the cost of treatment. Thus, the routine use of amoxicillin in post-adenotonsillectomy patients remains controversial. According to our results and data from the literature, no statistically significant difference was found for most of the post-adenotonsillectomy morbidity factors. Therefore, antibiotic therapy to mitigate post-op morbidity must be preceded by a careful analysis of the risks and benefits inherent to the use of antibiotics, being their use only recommended in selected cases where the advantages outweigh the known deleterious effects of antibiotic therapy.
